# Extracellular ATP release predominantly mediates Ca^2+^ communication locally in highly organised, stellate-Like patterned networks of adult human astrocytes

**DOI:** 10.1371/journal.pone.0289350

**Published:** 2023-10-03

**Authors:** Si Li, E. Scott Graham, Charles P. Unsworth

**Affiliations:** 1 Department of Engineering Science, The University of Auckland, Auckland, New Zealand; 2 The MacDiarmid Institute for Advanced Materials and Nanotechnology, Auckland, New Zealand; 3 Department of Molecular Medicine and Pathology & Centre for Brain Research, The University of Auckland, Auckland, New Zealand; Eötvös Loránd Research Network Biological Research Centre, HUNGARY

## Abstract

The ‘Astrocyte Network’ and the understanding of its communication has been posed as a new grand challenge to be investigated by contemporary science. However, communication studies in astrocyte networks have investigated traditional petri-dish *in vitro* culture models where cells are closely packed and can deviate from the stellate form observed in the brain. Using novel cell patterning approaches, highly organised, regular grid networks of astrocytes on chip, to single-cell fidelity are constructed, permitting a stellate-like *in vitro* network model to be realised. By stimulating the central cell with a single UV nanosecond laser pulse, the initiation/propagation pathways of stellate-like networks are re-explored. The authors investigate the mechanisms of intercellular Ca^2+^ communication and discover that stellate-like networks of adult human astrocytes *in vitro* actually exploit extracellular ATP release as their dominant propagation pathway to cells in the network locally; being observed even down to the nearest neighbour and next nearest neighbouring cells—contrary to the reported gap junction. This discovery has significant ramifications to many neurological conditions such as epilepsy, stroke and aggressive astrocytomas where gap junctions can be targeted. In cases where such gap junction targeting has failed, this new finding suggests that these conditions should be re-visited and the ATP transmission pathway targeted instead.

## Introduction

Over the past 10 years, scientific research has revealed that the astrocyte [1] possesses much more diversity in its functionality that was commonly thought beyond its subservient role to the neuron [2–4]. Such functionality has traversed endocannabinoid synthesis [5], immunological signalling [6–8], the production of cytokines and chemokines [9] and the unanticipated modulation of the synaptic cleft between neurons [10–12]. As a consequence of this newly discovered functionality, contemporary literature has proposed that there should be a drive to understand the ‘Astrocyte Network’ on an equal footing to that as the traditional ‘Neural Network’. This drive is in order to better understand the pathways that calcium (Ca^2+^) communication engages at the network level [13]. Such exploratory research could provide new knowledge and understanding of how astrocytes communicate in networks, revealing clues that would enable the development of novel strategies to treat a whole host of astrocyte related neurological disorders, such as in epilepsy [14, 15], stroke [16], focal cerebral ischemia and aggressive astrocytoma brain cancers such as glioblastoma multiforme [17] and diffuse intrinsic pontine gliomas [18]. Our group’s work is involved with adult human astrocytes and in this article, we will use the well understood human teratocarcinoma NTERA2/D1 cell line (also referred to as hNT/NT2) [19], which has been used for cell transplantation in stroke therapy [20], expresses universal neuronal/astrocytic markers [19], validated to be an authentic alternative to human primary neuronal cells [21] and provides a simple and convenient model of human neural tissue [22, 23], with no ethical concerns being derived from an immortalised stem cell line [19]. Our group has performed extensive work in the field of cell patterning, employing hNT astrocytes on parylene-C/SiO_2_ chips with the most relevant to this study, being the development of a highly organised single cell 10×10 grid network of hNT astrocytes to enable study of their communication accurately and repeatedly, being the largest single cell astrocyte networks of its kind [24]. In addition, we have demonstrated that Ca^2+^ responses can be evoked from hNT astrocytes when stimulated with nanosecond UV lasers by Raos *et al* [25]. In this article, we combine our work of laser stimulation of human astrocytes [25] with our cell patterned [26] organised grid networks of astrocytes to understand the mechanisms of communication that are evoked in networks of astrocytes. Specifically, we build highly organised 5 × 5 grid networks of hNT astrocytes on parylene-C/SiO_2_ chips and stimulate the central cell in the network with a nanosecond UV laser pulse. By applying pharmacological agents to block the common initiation and propagation pathways in astrocytes, we then proceed to determine the exact initiation and propagation pathways that are repeatedly engaged in astrocyte network Ca^2+^ communication in the inner and outer regions of the network.

Current research has only reported the initiation and propagation pathways for clusters or sub-clusters of unorganized monolayers of astrocytes of various species under electrical, mechanical and laser stimulation in networks as follows.

The initiation pathway: The mechanism for intracellular Ca^2+^ initiation was demonstrated to be induced either by extracellular Ca^2+^ or intracellular Ca^2+^ stores [25–29]. Venance *et al*. demonstrated that Ca^2+^ waves of primary rat astrocytes induced either by mechanical stimulation was dependent on the presence of external Ca^2+^ [29]. Zhao *et al*. targeted femtosecond laser pulses to the cell membranes of purified rat astrocytes and *hypothesised* that the pore allowed for a small influx of extracellular Ca^2+^ from the extracellular fluid resulting in a localized increase in intracellular Ca^2+^ [27]. However, Hill *et al*. observed that ATP elicited Ca^2+^ elevations in NT2 derived astrocytes were inhibited by the intracellular store depletory thapsigargin, suggesting that intracellular stores were the main Ca^2+^ stores for signalling in astrocytes [30]. Furthermore, our group, Raos *et al*. developed a nanosecond laser stimulation technique for elevating Ca^2+^ increases in NT2 derived astrocytes and observed that stimulation was still possible in absence of extracellular Ca^2+^ and thus, hypothesized that localized ablation of the ER was the primary factor in stimulating Ca^2+^ response by nanosecond lasers [25]. Most recently, Fujii *et al*. observed that both omitting extracellular Ca^2+^ and inhibiting Ca^2+^ stores did not affect Ca^2+^ increases in primary rat astrocytes by mechanical stimulation [31]. Fujii *et al*. demonstrated that mechanical stimulation may induce Ca^2+^ release from thapsigargin-insensitive Ca^2+^ stores.

The propagation pathways: Since astrocytes main physical connection is via intercellular gap junctions [31, 32], the mechanism for intracellular Ca^2+^ propagation via transmission of Ca^2+^ and inositol 1,4,5-trisphosphate (IP3) ions through gap junctions has become intensively studied [33–35]. Other studies involving primary rat astrocytes have demonstrated that Ca^2+^ waves can propagate between physically separated cells, indicating that purinergic signalling may also be involved in Ca^2+^ propagation, presumably by secreted extracellular adenosine triphosphate (ATP) [36–38]. ATP can bind to the membrane-bound receptors, resulting in the generation of IP3 which triggers the release of intracellular Ca^2+^ from Ca^2+^ stores. Guthrie *et al*. demonstrated that purinergic antagonists blocked propagation of electrically evoked Ca^2+^ waves, indicating that extracellular ATP was required for normal Ca^2+^ wave propagation in primary rat astrocytes [38]. Takano *et al*. demonstrated that Ca^2+^ waves of cortical murine astrocytes by mechanical stimulation could propagate to neighbouring astrocytes not only within the same confluent domain but also to neighbouring non-connected astrocyte areas [39]. Zhao *et al*. investigated how to use femtosecond lasers to elevate Ca^2+^ increases in rat astrocytes and demonstrated that femtosecond laser-induced Ca^2+^ waves were predominantly mediated by the extracellular messenger ATP in partial collaboration with gap junctions [27]. Most recently, Fujii *et al*. demonstrated that persistent Ca^2+^ increases propagated rapidly via gap junctions in the proximal region and transient Ca^2+^ increases propagated slowly via extracellular diffusion of ATP in the distal region in primary rat astrocytes [31]. In addition, Hill *et al*. investigated the functional properties of NT2 derived neurons and astrocytes and demonstrated that mechanically-induced Ca^2+^ waves of NT2 derived astrocytes were gap junction and purinergic signalling dependent [30]. This was supported by inhibition of Ca^2+^ responses in the presence of both gap junction blocker and purinergic receptor antagonist.

Previous studies in astrocytes have been limited to clusters or sub-clusters of unorganized astrocyte monolayers [29, 30, 36, 38–40]. To reproduce the morphology and connections of astrocytes *in vitro* and to simplify the complexity of communication *in vitro* cultures, it is important to control where the cells contact with one another. The ability to culture astrocytes in a controlled network configuration with prescribed cell separation distances and connectivity would give insight into the response of astrocyte networks, in a scale that is more physiologically relevant than previously possible. Previous *in vitro* semi-organized network platforms demonstrated by Jordan *et al*. patterned the astrocytes in a trench grid network [41]. Most recently, our group established an experimental platform that can accurately organise astrocytes within a single cell 10×10 grid array network on parylene-C/SiO_2_ substrates [23, 41]. The majority of the astrocyte bodies resided on the nodes and the astrocytes were connected to neighbouring cells through the narrow lines between the nodes. In this work, we combine our work on organized astrocyte networks with our work on nanosecond UV lasers stimulation, which successfully elevates Ca^2+^ releases in hNT astrocytes, to investigate the underlying initiation and propagation mechanisms of intracellular Ca^2+^ waves in organized regular grid astrocytic networks. Specifically, we focus on (1) intracellular Ca^2+^ storage, (2) influx of extracellular Ca^2+^, (3) gap junction communication, and (4) ATP pathway contributions in astrocytic networks.

## Materials and methods

### Astrocyte grid networks design & fabrication

**Fig 1:**
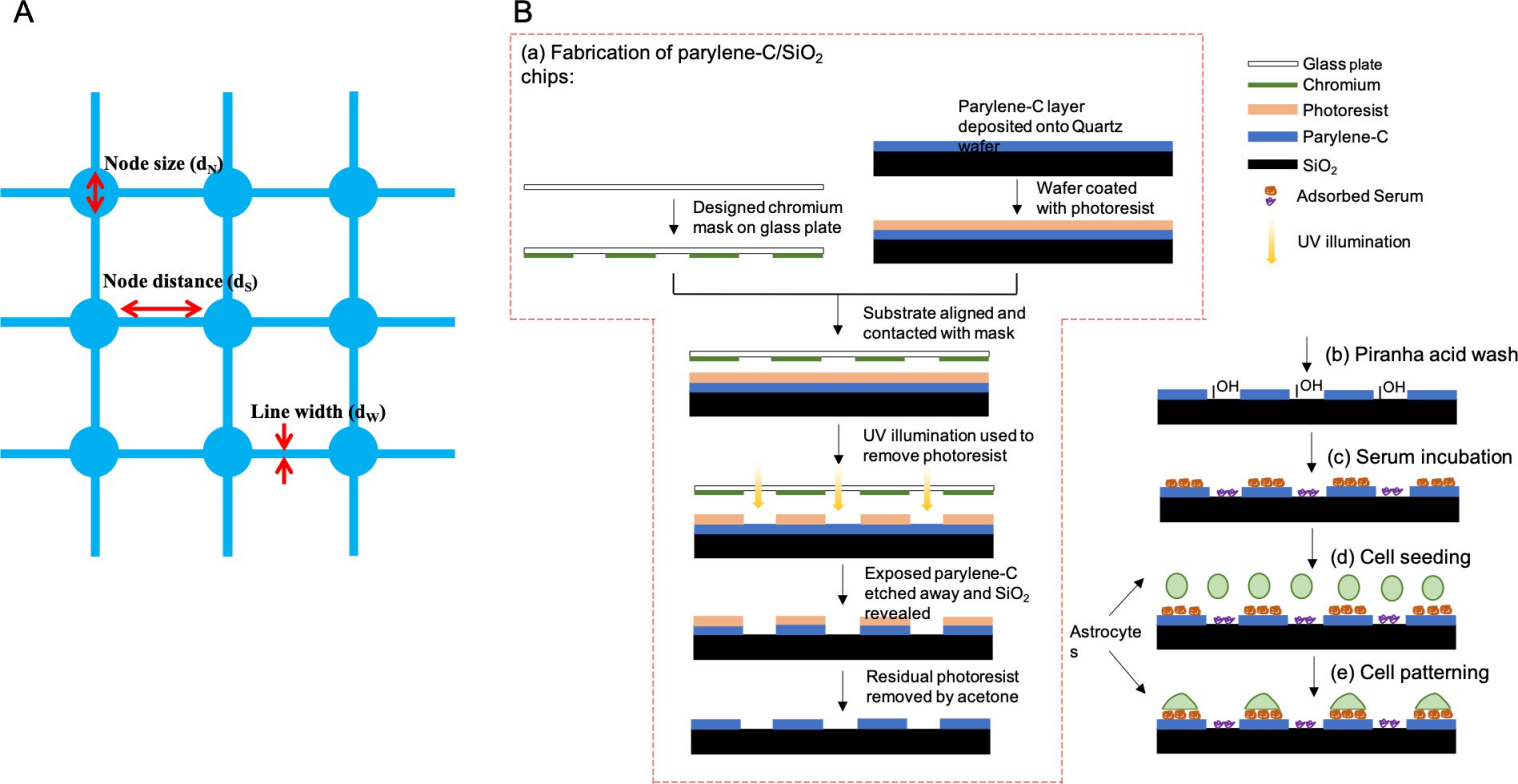
(A) Design of astrocyte grid networks. Circular parylene-C nodes connected by narrow lines. The three optimal parameters were: 65μm node size (d_N_), 90μm node distance (d_S_) and 5μm line width (d_W_) are indicated [11]. (B) Flow diagram of (a) the photolithography process for fabrication of parylene-C/SiO_2_ chips, (b) chip cleaning, (c) serum incubation, (d) cell seeding, and (e) cell patterning. Piranha acid wash cleaned off organic residues and debris during the fabrication. Serum incubation coated chips, promoted cell adhesion on parylene and inhibited cell adhesion on SiO_2_. After the cell seeding, the astrocytes on the parylene area attached to the surface and were repelled from the SiO_2_ substrates.

Here we use the method of, Li *et al*., where hNT astrocytes were patterned on the biomaterial, parylene-C, to high resolution at the single-cell level on nodes in a regular grid array (Fig 1) [24, 42]. We used parameters of d_N_ = 65 μm node size, d_W_ = 5 μm interconnecting line width, and d_S_ = 90 μm node distance which Li *et al*. demonstrated achieved optimally defined single-cell grid networks.

Photolithography was used to construct the parylene-C grid networks on parylene-C/SiO_2_ substrates for hNT astrocytes as in [24, 42]. A parylene-C layer was deposited onto a quartz wafer via chemical vapour deposition (CVD). Positive photoresist was then spin-coated on the wafer using a suitable photo-resist coating system. Substrates were aligned with pre-manufactured chromium photomask with the network design and then exposed with a UV illumination to remove exposed photoresist. Any unprotected parylene area was then etched off by an oxygen plasma etch system to reveal the underlying SiO_2_. Wafers were then diced into chips, rinsed in deionized H_2_O and blown dry with nitrogen. The residual photoresist layer was removed by rinsing with acetone. The manufactured parylene-C/SiO_2_ chips were then cleaned with piranha acid in a 5:3 ratio of 30% hydrogen peroxide (H_2_O_2_) and 98% sulphuric acid (H_2_SO_4_) for 10–15 min and rinsed in Milli-Q water. After the piranha acid wash, chips were immediately placed in a 24-well plate and sterilized in 2% Penicillin-Streptomycin-Glutamine (PSG) solution for 30–60 min at room temperature. Finally, the chips were incubated in fetal bovine serum (FBS) for 3 h at 37°C and 5% CO_2_ to activate the patterns.

### hNT astrocyte cell-culture

hNT astrocytes were differentiated from the NTera2/D1 (NT2/hNT) cell line over 9 weeks according to the protocols [19, 22, 29] hNT precursor cells were cultured in 10% FBS DMEM: F12 media containing 10 μM retinoic acid (RA) in petri dish, replating every 2–3 days for the first 2 weeks. Then, cells were transferred into T-75 flasks with media changes every 2–3 days treated with 10 μM RA for 7–10 days. At week four, cells with neuronal morphology were harvested by selective trypsinization and removed from the culture. The remaining underlying astrocyte precursor cells were replated in the T-75 flask and cultured in 5% FBS/DMEM: F12 with decreasing concentrations of the mitotic inhibitors (uridine (Urd), 5-fluoro-2’-deoxyuridine (FUdR) and β-D-arabinofuranoside (AraC)) for further 5 weeks, with media changes every 2–3 days. The NT2 astrocytes were finally harvested with 0.05% Trypsin and seeded on parylene-C/SiO_2_ chips.

hNT astrocytes were dissociated from the flask with trypsinization, rinsed with 5% FBS/DMEM: F12 and centrifuged at 200 g for 5 min. Then, astrocytes were re-suspended in 1mL of the medium, counted and seeded on parylene-C/SiO_2_ chips at a density of 50 cells/mm^2^ [40]. The chips were incubated at 37°C and 5% CO_2_ for 2 days to allow the astrocytes to migrate onto the patterns (Fig 1).

### Laser stimulation

Nanosecond laser pulses were generated by a MicroPoint Laser Illumination and Ablation System (Andor Technology) connected to an Olympus BX53 upright microscope and controlled by Andor iQ software. The microscope was set up to operate in reflection mode with 10× water immersion objective. The laser system consisted of a pulsed nitrogen laser producing single 200 μJ, 3 ns duration pulses as determined optimal to induce Ca^2+^ elevations in hNT astrocytes [25]. The wavelength of the laser was tuned from its standard wavelength of 340 nm to the optimum 365 nm wavelength required to induce Ca^2+^ elevations in hNT astrocytes [25] by a dye cell containing 6 mM 2-(4-Biphenylyl)-5- (4-t-butylphenyl)-1,3,4-oxadiazol (BPBD) in methanol. The optical path contained a mirror galvanometer which facilitated fast and precise steering of the laser beam to user-specified locations and a motorized attenuator that allowed for selective attenuation of the beam energy. Laser pulses were manually triggered.

### Cell labelling & chemical blockers

hNT astrocytes were stained with a live 1 μM Cell Tracker Green Dye (CMFDA) and Hoechst 33342 (Blue) at two drops per mL in 5% FBS/DMEM: F12 for 30 min at 37°C and 5% CO_2_. For intracellular Ca^2+^ measurements, cells were stained with a live 1 μM Fluo-4 AM dye and Hoechst 33342 (Blue) for 40–60 min at room temperature. Cells were rinsed in FluoroBrite with 1% FBS twice to improve signal-to-noise ratio of fluorophores and preserve cell health.

Various pharmacological blocking agents were administered to the hNT astrocytes before the experiments in order to identify the initiation and propagation pathways mechanisms of hNT astrocytes. The initiation and propagation pathways were blocked as follows:

1. **ER Ca**^**2+**^ **store:** Thapsigargin (Tg) has been shown to deplete the Ca^2+^ store inside the endoplasmic reticulum (ER) of hNT astrocytes manufactured by Hill *et al*. [30]. Thus, according to [18] the astrocytes were incubated in 1 μM thapsigargin medium for 30 min before and during the experiments.
2. **Extracellular Ca**^**2+**^: Ca^2+^free DMEM and Ca^2+^free HBSS were demonstrated to deplete extracellular Ca^2+^ in hNT astrocytes [25]. Thus, Ca^2+^free DMEM/HBSS was used to replace the regular medium after the Fluo-4 AM loading. This treatment was used to remove the extracellular Ca^2+^ sources immediately before the laser stimulation experiments.
3. **Gap junction:** Carbenoxolone (CBX) has been demonstrated to block the gap junction pathways in hNT astrocytes [30]. Thus, the hNT astrocytes were incubated in 100 μM CBX medium and remained in medium during the laser stimulation experiment.
4. **Extracellular messenger ATP:** Suramin has been reported to be a non-selective antagonist of purinergic receptors and used in primary rat astrocytes [26, 30]. We found that by supplying 100 μM suramin in the medium for 30 min before and during the experiments behaved the same for *in vitro* hNT astrocytes.

### Cell imaging & image processing

Fluo-4 fluorescence images were captured by an Andor Clara-E interline CCD camera that was controlled by the Andor iQ software package. The Fluo-4 fluorescence images were imported into the image processing software ImageJ ©. The region of interest (ROI) of each cell was created manually by the ImageJ’s ROI management toolbox and the fluorescence of each ROI during every frame was measured automatically. The fluorescence data was then processed in MATLAB © (R2016b Pro, The MathWorks Inc., Natick, MA) to determine the change in fluorescence over the baseline fluorescence prior to stimulation (ΔF/F_0_).

### Statistical analysis

All quantitative data in the text and figures are presented as mean ± standard deviation. Statistical analysis of the results was performed using the software GraphPad Prism 7 and 5% and 10% significance levels are presented using a student two-sided t-test.

## Results

### Cell patterning in organised grid network

**Fig 2:**
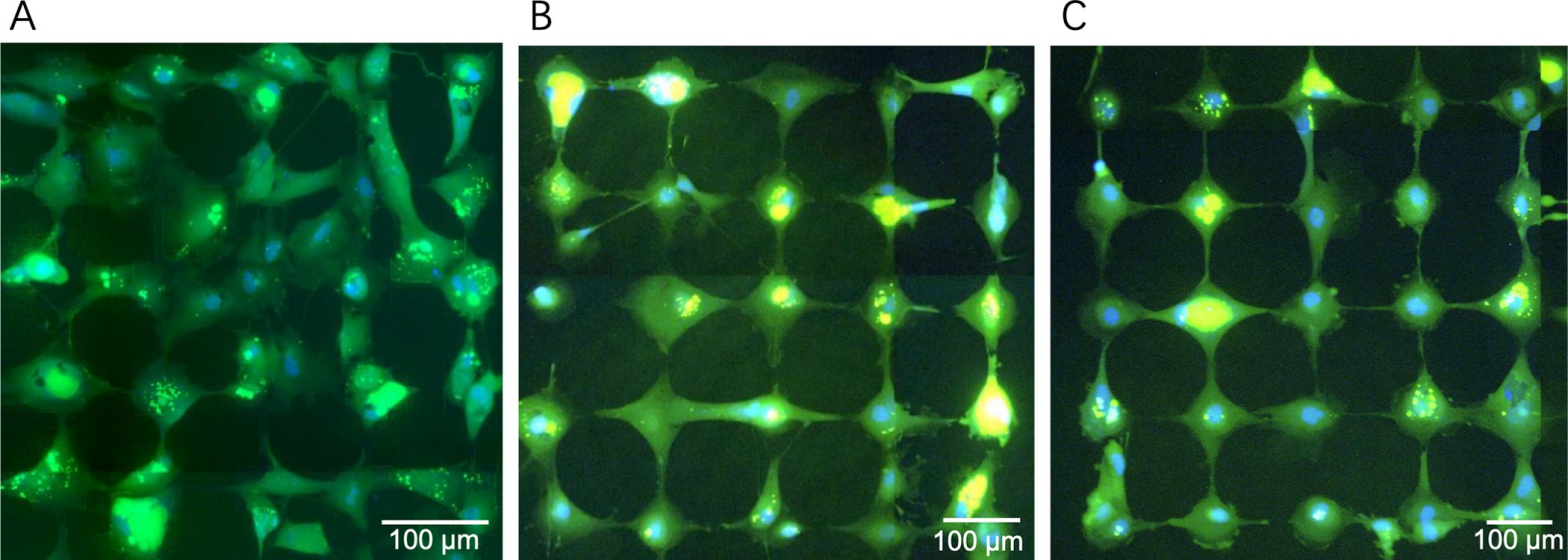
Patterned astrocyte networks live stained with CMFDA and Hoechst 33342. The networks had node size of 65 μm, interconnecting line widths of 5 μm, and (A) 60-μm inter-node distance, (B) 75-μm inter-node distance, and (C) 90-μm inter-node distance, respectively. Scale bar = 100 μm.

We demonstrated that hNT astrocytes could be successfully patterned on organised grid networks with optimal network parameters being a 65-μm node size, 5-μm line width, and 90-μm node distance (65/5/90) [12], we showed that 80% of astrocyte bodies were contained as single cells on the nodes of the grid networks and connected via the tracks to astrocytes on other adjacent nodes. In order to determine the effect of inter-node distance on Ca^2+^ propagation through the networks, the node size and line width of the networks were set at 65 μm and 5 μm, respectively, and the inter-node distances were varied (60 μm, 75 μm and 90 μm) where 90 μm was the maximum field of view using the laser ablation system which permitted a (5 × 5) grid network. Fig 2, shows the patterning obtained for hNT astrocytes at 60, 75 and 90 μm node distances networks. As can be seen, networks with 60 μm inter-node distance had disrupted patterning results (Fig 2A), and good patterning obtained for networks with 75-μm node distance, and 90-μm node distance as predicted by [24]. Considering that we would stimulate the central cell in the network, we elected to show that Ca^2+^ propagation from the central cell through to adjacent cells (the nearest neighbours NI) and the outer cells (the next nearest neighbours NII). Additionally, inter-node distances greater than 90 μm were associated with network magnifications of less than 5 × 5, which would not be sufficient to examine the Ca^2+^ propagation in the networks of at least the nearest neighbours and next nearest neighbours. Thus, networks with a 65-μm node size, 5-μm interconnecting line width, and a node-distances of 75 or 90 μm (Fig 2B and 2C) were used to investigate the effect of different inter-node distances on Ca^2+^ propagation through the networks.

### Determining the optimum laser power for calcium signalling in astrocytes networks

**Fig 3:**
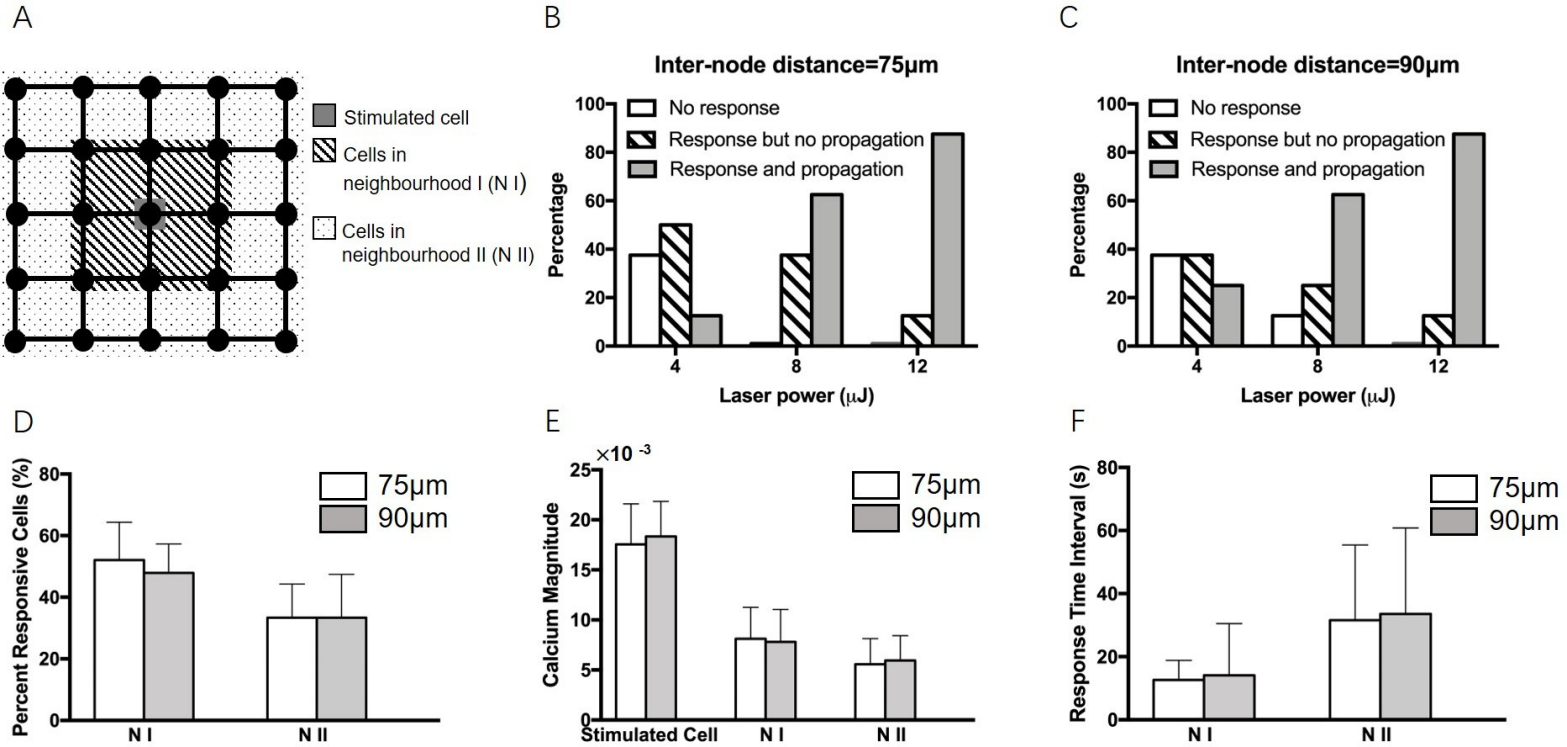
(A) Cell classification into three groups according to the distance from the laser-stimulated cell: Stimulated cell (grey), cells in neighbourhood N I (hatching), cells in neighbourhood N II (dotted). Different laser power generated different Ca^2+^ response in both (B) 75 μm and (C) 90 μm node distances networks. Experiment repeats equal to 8 (N = 8).’No response’ refers to cells with no observed Ca^2+^ increase (white). ‘Response but no propagation’ refers to cells giving a Ca^2+^ response, but Ca^2+^ waves did not propagate to neighbouring cells (hatching). ‘Response and propagation’ indicates cells that produced Ca^2+^ responses and Ca^2+^ waves propagated to the neighbouring cells in the network (grey). (D) Percentage of responsive cell, (E) Ca^2+^ magnitude of both stimulated cell and neighbouring cells, and (F) cell response time interval for both 75 μm and 90 μm node distances networks. Astrocytes were stimulated by a single laser pulse of 12 μJ in (D-F). Error bars represent the standard deviation (N = 6).

The neighbouring hNT astrocytes in the organised networks were classified into two regions according to their distance from the stimulated central cell as follows: cells in the inner neighbourhood (N I)–namely the nearest neighbours directly adjacent to the surrounding the central cell, and cells in the outer neighbourhood (N II)—namely the next nearest neighbours directly adjacent to the inner neighbourhood, as shown in Fig 3A. Whilst Raos *et al*. [25] determined that 4–12 μJ, maintaining a cell viability of 87.5% for single hNT astrocytes on nodes, we determined that a 12 μJ laser pulse was optimum to evoke Ca^2+^ propagation from the central cell to the outer neighbouring cells in 5×5 network for both 75 and 90μm inter-node distances [43], shown Fig 3B and 3C. It was found that there was no significant difference in the results between inter-node distances of 75 and 90 μm.

In addition, we observed that when the astrocyte in the centre of the network was stimulated by a single laser pulse of 12 μJ, the intracellular Ca^2+^ signals propagated from the stimulated astrocyte to the adjacent cells (namely, the nearest neighbours) in the patterned network and then to the outer cells (namely, the next nearest neighbours) in the patterned network. Fig 3D–3F shows the results of the percentage of responsive cells, Ca^2+^ magnitude of both stimulated cell and neighbouring cells, and cell response time interval for both 75 μm and 90 μm node distances networks, respectively. Ca^2+^ magnitude refers to the maximum value of the fluorescence change (ΔF/F_0_) in Ca^2+^ traces.

It was found in Fig 3D that the percentage of responsive cells in neighbourhood II (N II) was lower than the closer cells in neighbourhood I (N I) and there was no significant difference in the responsive percentage in N I and N II between 75 μm and 90 μm node distances. In Fig 3E, we found that the magnitudes of the Ca^2+^ responses of nearest neighbours (in the first neighbourhood) was reduced by a factor of 2.13 from the central stimulated cell and the Ca^2+^ responses of next nearest neighbours (in the second neighbourhood) was reduced by a factor of 3.41 from the central stimulated cell. It was found that there was no significant difference in the Ca^2+^ magnitude between 75 μm and 90 μm node distances networks in either the N I or N II neighbourhoods. Furthermore, we observed that the mean response time interval of cells in N I was 17.3±16.25 s and the mean response time interval of cells in N II was 36.4±28.2 s. No significant difference of response time was observed between 75 μm and 90 μm node distances networks in either N I or N II.

### Ca^2+^ responses in grid organised network in different groups

**Fig 4:**
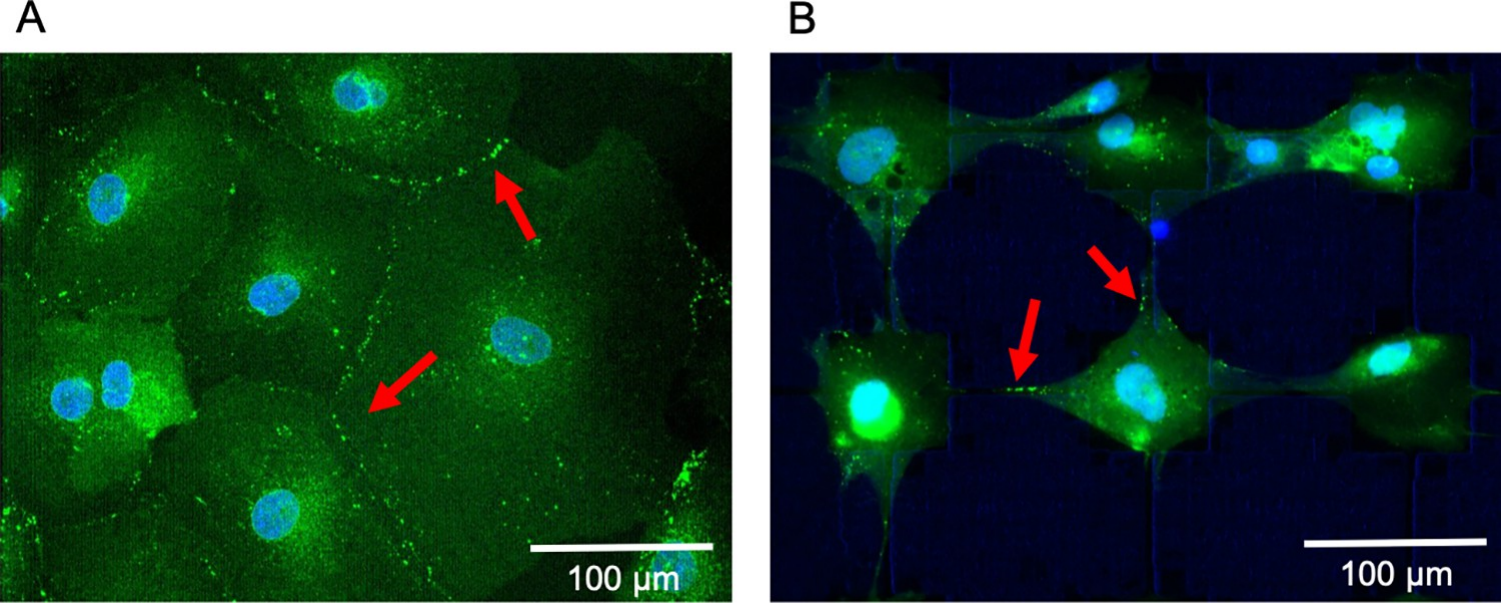
Connexin 43 (Cx43) staining of hNT astrocytes in: (A) petri dishes, and (B) organised grid networks. Arrows show areas of the Cx43 staining between adjacent cells, suggesting gap junction formation.

We first stained the Cx43 gap junction protein to visually observe the amount of gap junctions that exist in standard non-patterned *in vitro* culture of hNT astrocytes as compared to organised networks of hNT astrocytes. Fig 4 shows the immunofluorescence staining of the Cx43 gap junction protein for unorganised hNT astrocytes in petri dishes (Fig 4A) and organised hNT astrocytes in grid networks (Fig 4B). In Fig 4A, it was observed that the Cx43 staining at the cell circumference suggests that the gap junctions were formed along the cytoplasmic peripheries of the cells. However, in Fig 4B, the Cx43 staining occurred at the ends of cell processes suggested that gap junctions were formed between cells processes in the patterned astrocyte networks. Secondly, as the astrocyte architecture formed into the networks it was observed that the gap junction localisation is considerably lower than that those observed in non-patterned astrocytes.

**Fig 5:**
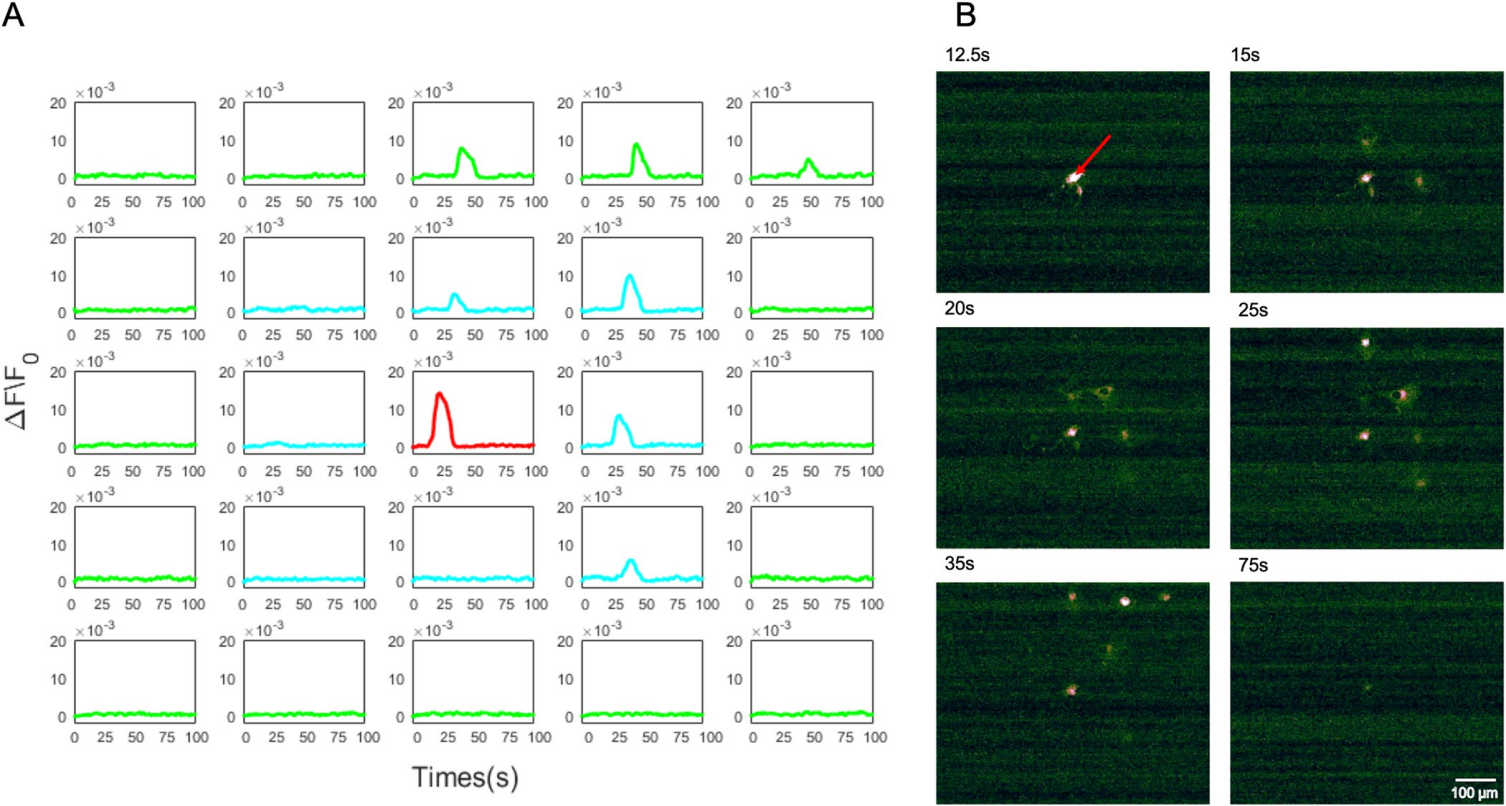
A spatial arrangement of the single Ca^2+^ traces of each hNT astrocyte in the 5×5 grid network in control group, without any pharmacological agents: Stimulated cell (central—red), N I (blue), and N II (green). Ca^2+^ responses are represented as the change in fluorescence over the baseline fluorescence prior to stimulation (ΔF/F_0_).

In order to analyse the results of pharmacological blocking agents, cells that were not treated with any pharmacological agents were used first as the control group. The central astrocyte in the network was stimulated using a single 12 μJ laser pulse. The Ca^2+^ intensity of the laser-stimulated cell increased dramatically in response to the stimulation. Further, intracellular propagation of Ca^2+^ to the outer neighbourhoods of the network was also observed [43]. Finally, the Ca^2+^ concentration decreased back to the baseline level. It was observed that the propagation of the intercellular Ca^2+^ is not equal in every direction, which was different from the mathematical models demonstrated by *Lallotuette et al*. [44]. Fig 5 shows the Ca^2+^ responses of the stimulated cell, the astrocytes in the neighbourhood N I and N II of the organised grid network in control group. Preliminary observations of simple bulk Ca^2+^ flow in a grid network of hNT astrocytes stimulated by a UV laser pulse was reported by Li *et al*. [43] at conference level.

We then proceeded to investigate the Ca^2+^ initiation mechanism of the central stimulated cell for the hNT astrocyte. The mechanisms underlying the Ca^2+^ initiation was pharmacologically examined by treating cells with 1 μM Tg or Ca^2+^-free DMEM/HBSS. Tg inhibits Ca^2+^ storage in the ER, while the use of Ca^2+^-free DMEM/HBSS was used to inhibit Ca^2+^ influx from the medium.

**Fig 6:**
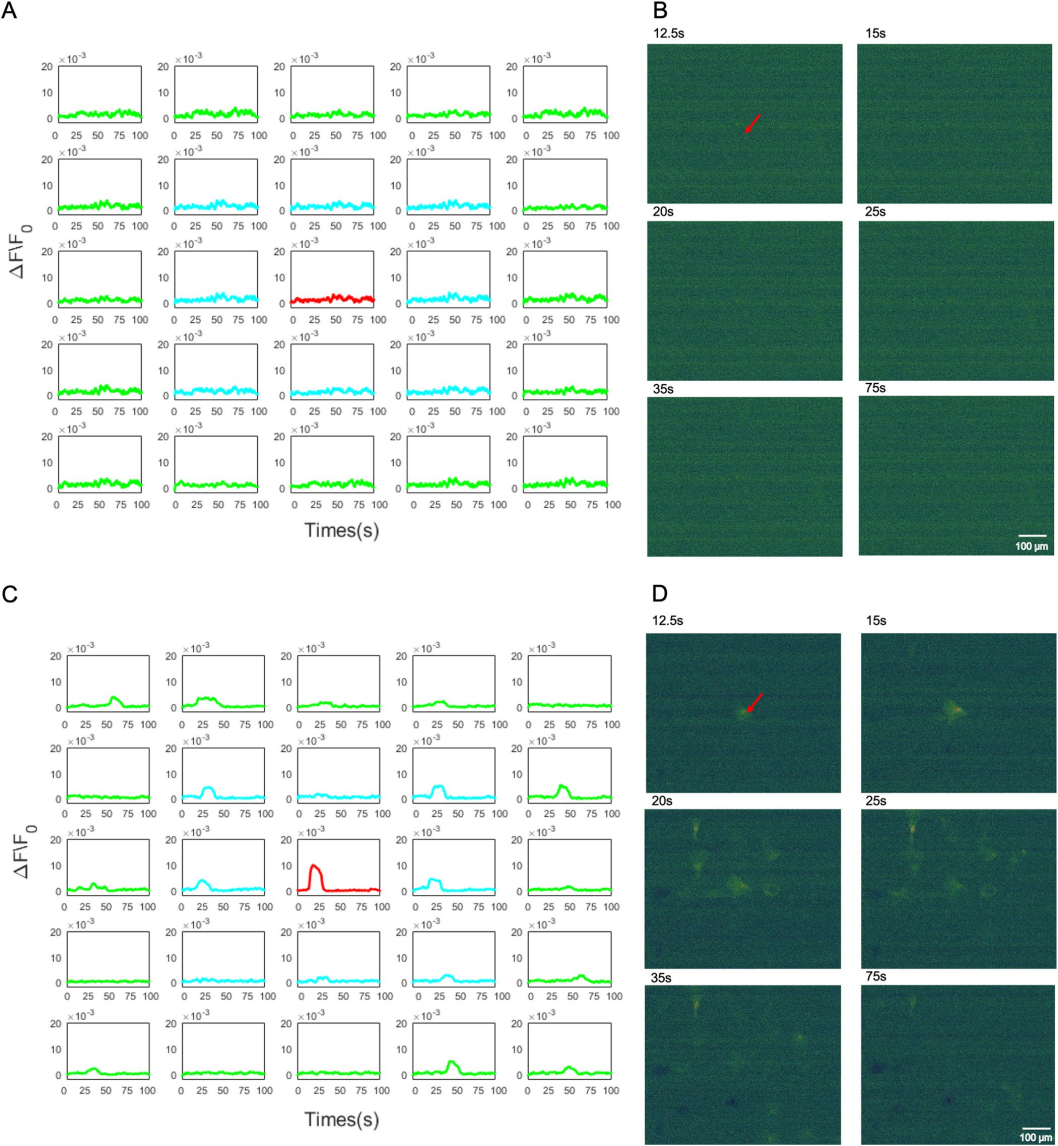
(A) A spatial arrangement of the single Ca^2+^ traces of each hNT astrocyte in the 5×5 grid network treated with Tg, which depleted ER calcium store: stimulated cell (central—red), N I (blue), and N II (green). (B) Pseudo-colour Fluo-4 fluorescence images showing the propagation of an intercellular Ca^2+^ waves subsequent to the nanosecond laser stimulation of Tg group. (C) A spatial arrangement of the single Ca^2+^ trace of each hNT astrocyte using Ca^2+^-free DMEM/HBSS which initially removes extracellular Ca^2+^ from the medium: stimulated cell (red), N I (blue), and N II (green). (D) Pseudo-colour Fluo-4 fluorescence images showing the propagation of an intercellular Ca^2+^ waves subsequent to the nanosecond laser stimulation of Ca^2+^-free group. Ca^2+^ responses are represented as the change in fluorescence over the baseline fluorescence prior to stimulation (ΔF/F_0_).

1. **Intercellular Ca**^**2+**^ **of the ER blocked using Tg.** In Fig 6A and 6B, we observed that Tg successfully blocked the intracellular Ca^2+^ in the stimulated cell as no fluorescence was observed in the central laser stimulated cell. Therefore, no Ca^2+^ responses were observed in the NI and N II neighbourhoods.
2. **Extracellular Ca**^**2+**^ **from media blocked using Ca**^**2+**^**-free DMEM/HBSS.** In Fig 6C and 6D, shows the Ca^2+^ responses with the presence of Ca^2+^ free media. We observed that the stimulation was still possible in the absence of extracellular Ca^2+^, which were consistent with the results reported by Raos *et al*. [25]. A Ca^2+^ responsive peak in the stimulated cell was observed immediately after the laser stimulation. Further, the Ca^2+^ peak magnitude of astrocytes in neighbourhoods NI and NII to be much lower than that observed in the stimulated cell.

Next, we investigated the mechanism by which Ca^2+^ propagation occurs from the central stimulated cell to neighbouring cells. The mechanisms underlying the Ca^2+^ propagation during Ca^2+^ waves were pharmacologically examined by treating cells with 100 μM CBX and 100 μM suramin, to block the gap junction communication and the extracellular ATP pathway, respectively.

**Fig 7:**
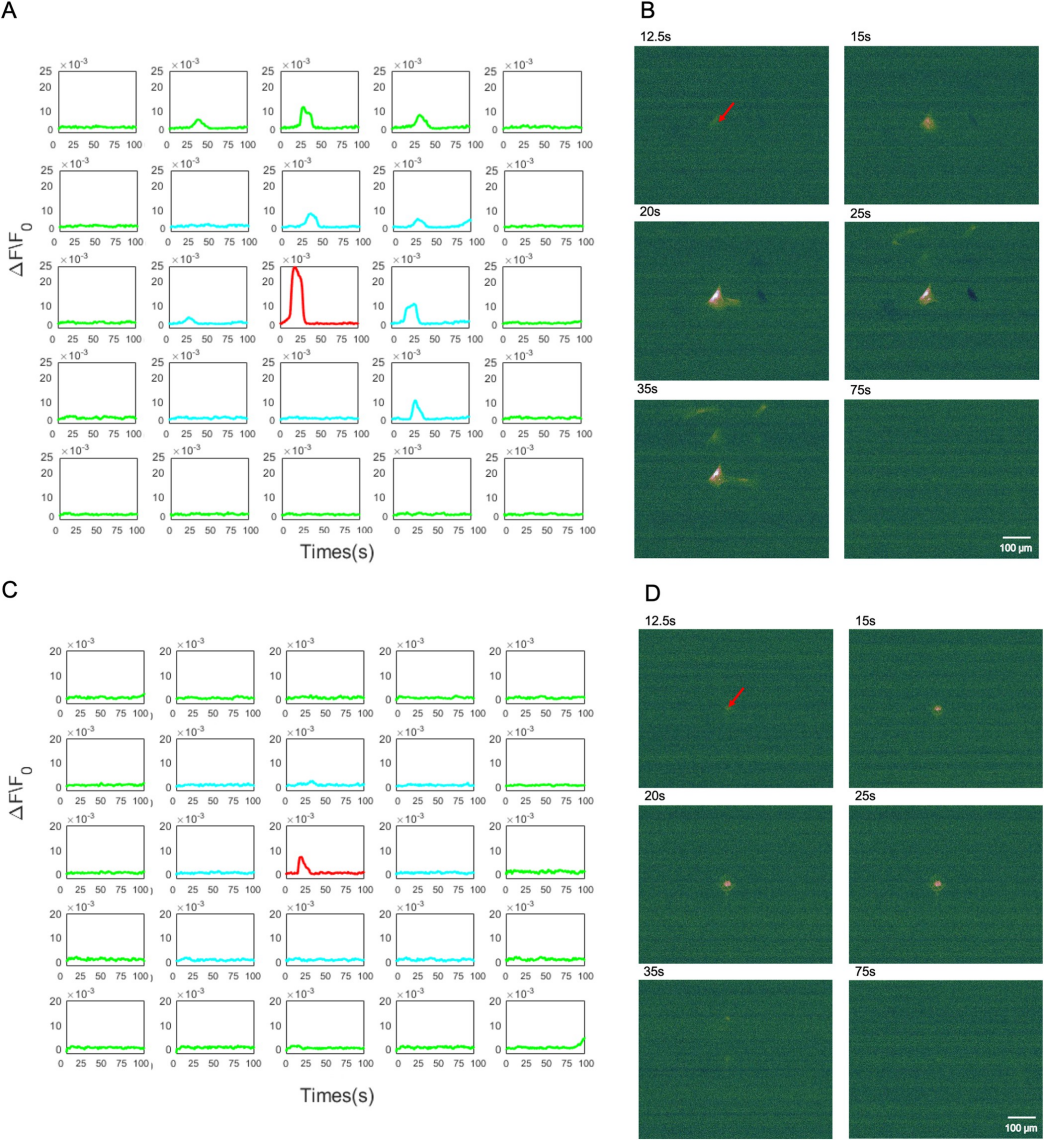
(A) A spatial arrangement of the single Ca^2+^ traces of each hNT astrocyte in the 5×5 grid network treated with CBX, which blocked gap junction communication: stimulated cell (central—red), N I (blue), and N II (green). (B) Pseudo-colour Fluo-4 fluorescence images showing the propagation of an intercellular Ca^2+^ waves subsequent to the nanosecond laser stimulation of Tg group. (C) A spatial arrangement of the single Ca^2+^ trace of each hNT astrocyte using suramin to block the extracellular ATP pathway: stimulated cell (red), N I (blue), and N II (green). (D) Pseudo-colour Fluo-4 fluorescence images showing the propagation of an intercellular Ca^2+^ waves subsequent to the nanosecond laser stimulation of Ca^2+^-free group. Ca^2+^ responses are represented as the change in fluorescence over the baseline fluorescence prior to stimulation (ΔF/F_0_).

1. **Gap junction communication blocked using CBX.** In Fig 7A and 7B, we observed that the Ca^2+^ propagation still occurred in the absence of the gap junction pathway.
2. **Extracellular ATP pathway blocked using suramin.** Fig 7C and 7D shows the Ca^2+^ responses of the stimulated cell and cells in neighbourhoods N I and N II when the extracellular ATP pathway was blocked by suramin. We observed the Ca^2+^ responses of astrocytes in neighbourhoods N I and N II to be severely inhibited due to the absence of the ATP pathway, with only one cell provided a heavily attenuated fluorescence in neighbourhood N I. No fluorescence was observed in N II.

### Analysis of Ca^2+^ initiation and propagation mechanism in organised networks

**Fig 8:**
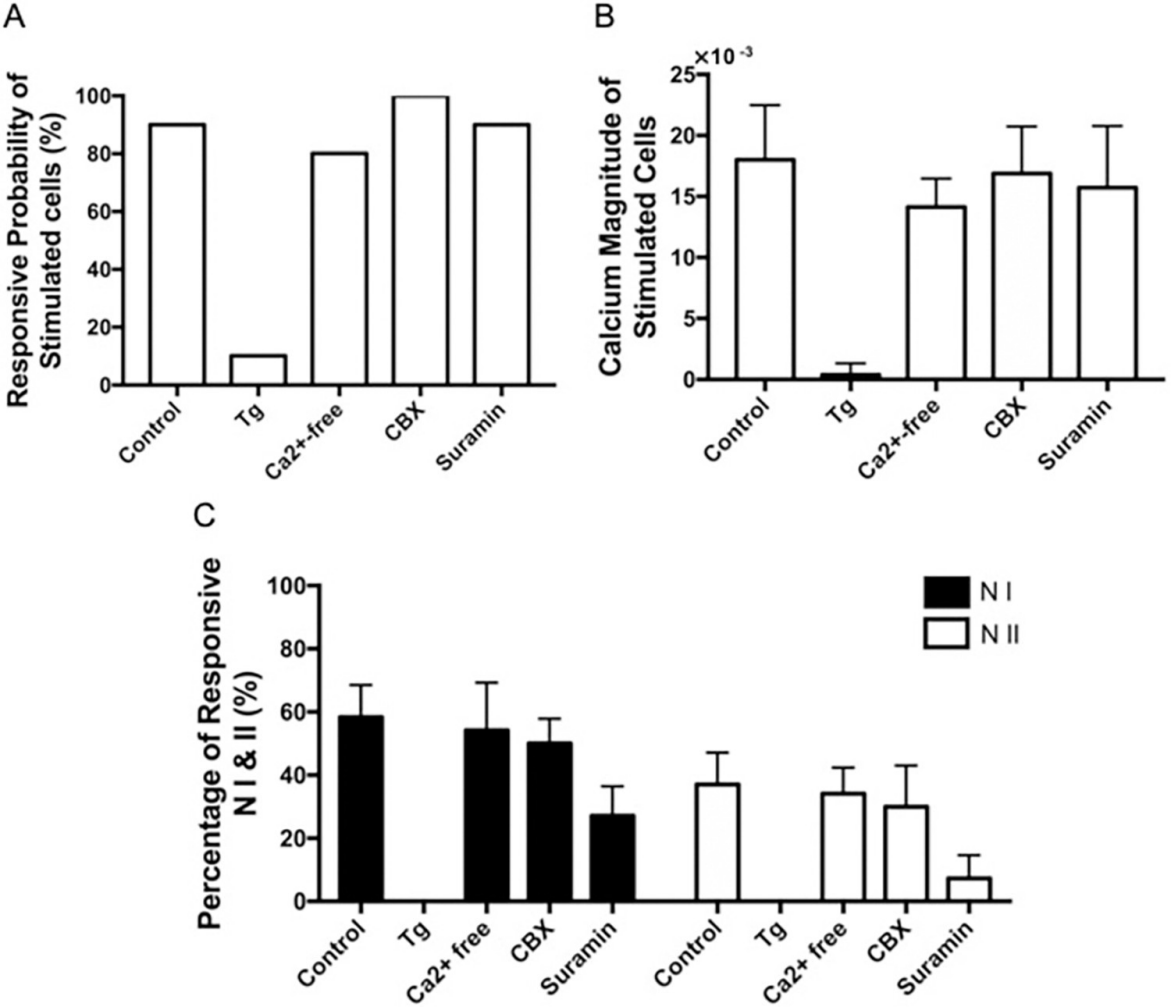
Pharmacological characterization of Ca^2+^ initiation and propagation. (A) Responsive probability of stimulated cells in control and different pharmacological agent groups. (N = 10) (B) Peak Ca^2+^ magnitude of stimulated cells in control and different treatment groups. (N = 6). (C) Percentage of total responsive neighbouring cells. (N = 6) (D) The percentage of responsive N I (cells in neighbourhood I from the stimulated cell) and N II (cells in neighbourhood II from the stimulated cell) in different groups. (N = 6). Astrocytes were stimulated by a single laser pulse of 12 μJ. Astrocytes were treated with 1 μM Tg, Ca2+-free DMEM/HBSS, 100 μM CBX, and 100 μM suramin. Error bars represent the standard deviation.

To investigate the mechanism of Ca^2+^ initiation, the percentages of responsive stimulated central cells in the different pharmacological groups (N = 10 experiments) were first calculated, as shown in Fig 8A. Fig 8A shows that for 9 out of the 10 experiments (90%) of the control group, the stimulated single central cell responded to laser stimulation. It was determined that, only in 1 out of the 10 experiments (10%) of Tg group, the stimulated central cell successfully responded to the laser stimulation. Therefore, the Ca^2+^ responses induced by the laser activation of the central astrocyte were abolished in the group treated with Tg (p<0.0001). It was found that in 8 out of 10 experiments (80%) of Ca^2+^-free group, the stimulated central cells responded to the stimulation. It was found that the Ca^2+^-free group did not have a significant difference compared to the control group (p = 0.556). We found that there was a 100% stimulation response in astrocytes treated with CBX, which implied that initiation did not require gap junctions. Further, there was a 90% stimulation response with the treatment of suramin. From this, it was determined that the CBX (p = 0.3306) and suramin (p = 0.99) groups were not significantly different to the control group.

The peak Ca^2+^ magnitudes of stimulated central cells in each pharmacological group were then calculated. Fig 8B shows the Ca^2+^ magnitudes of stimulated central cells in different pharmacological groups. The peak Ca^2+^ magnitude of stimulated central cells in the control group was (ΔF/F_0_ = 18±1.836 × 10^−3^). The peak Ca^2+^ magnitude of the stimulated central cell in Tg group (0.3833 × 10^−3^) was significantly lower than the control group (p = 0.0002). It was observed that the Ca^2+^ magnitude of stimulated central cells in groups with the treatment of Ca^2+^-free DMEM/HBSS was 14.13±0.9514 × 10^−3^ (p = 0.091). In addition, the Ca^2+^ magnitude of the stimulated central cells in the groups that were treated with CBX and suramin were 16.88±1.574 × 10^−3^ and 15.72±2.067 × 10^−3^, respectively. These results were also not significantly different to the control group (CBX: p = 0.6542; Suramin: p = 0.4282).

To investigate the mechanism of Ca^2+^ propagation, the percentages of responsive cells of N I and N II were separated and compared in different pharmacological groups as shown in Fig 8C. In the control group, a total of 58.83±10.2% of N I and 37±10.1% of N II cells showed Ca^2+^ responses. As we described above, there were no responsive cells in the N I and N II in the Tg group, given that there was no Ca^2+^ response in the stimulated cell. The percentages of responsiveness in the N I and N II in the Ca^2+^-free group were 54.17±15.1% (p = 0.5884) and 34.13±8.3% (p = 0.6017). However, these values were not significantly different to the control. It was observed that 50±7.9% of N I (p = 0.1449) and 30±13.1% of N II (p = 0.0326) were responsive in CBX group, respectively. It was found that only 27±9.4% cells in N I (p = 0.0003) and 7.3±7.3% cells in N II (p = 0.0002) responded in suramin group. This response was significantly lower than that recorded in the control group. In addition, we also observed that the percentage of responsive N II cells was significantly lower than that of N I cells.

In conclusion, these results indicated that the Ca^2+^ released from the Ca^2+^ store of the ER, blocked by Tg, is responsible for Ca^2+^ initiation in hNT astrocytes under the nanosecond laser stimulation, instead of the influx of extracellular Ca^2+^, and the ATP pathway, blocked by suramin, is responsible for Ca^2+^ propagation instead of the gap junctions in organised astrocyte networks.

## Discussion

Increases in Ca^2+^ concentration that spread from cell to cell provide a mechanism for astrocytes to coordinate many cellular activities. In this study, we investigated the Ca^2+^ initiation mechanisms and Ca^2+^ propagation mechanism from stimulated cell to neighbouring cells by UV nanosecond laser stimulation, in organised hNT astrocyte grid networks for the first time.

### Calcium initiation

In order to determine which pathway was responsible for Ca^2+^ initiation in hNT astrocytes, we examined the effect of removing the Ca^2+^ in the extracellular media (by using a Ca^2+^-free media) in order to halt Ca^2+^ influx intercellularly, which Raos *et al*. [25] used for hNT astrocytes *in vitro* cultures under UV nanosecond laser stimulation. And we employed Thapsigargin (Tg) to deplete the intercellular ER Ca^2+^ store, which Hill et.al [30] used on hNT astrocytes *in vitro* cultures under ATP stimulation. These results demonstrated that Ca^2+^ increases in hNT astrocytes stimulated by a UV nanosecond laser pulse were mainly dependent on intracellular ER Ca^2+^ storage instead of the extracellular Ca^2+^. This result was found to be similar to Hill *et al*.’s work [30] on hNT astrocytes in unorganised standard *in vitro* cultures albeit under ATP stimulation.

The closest laser stimulation regime to evoke Ca^2+^ responses was by Zhao *et al*. [45] who targeted IR femtosecond laser pulses to the cell membranes of rat primary astrocytes and observed the transient appearance of a pore in the cell membrane. Zhao *et al*. hypothesised that the pore allowed for the influx of extracellular Ca^2+^ into the cytoplasm resulting in a localised increase in intracellular Ca^2+^, which is then propagated throughout the stimulated cell by calcium induced calcium release (CICR). However, absorption dynamics are different between femtosecond and nanosecond laser ablation. Nanosecond laser pulses are less confined in the z-axis directions. This implies that a single nanosecond laser pulse could cause both the stimulation of the cell membrane and stimulation of the ER. Most recently, Raos *et al*. [25] demonstrated that single hNT astrocytes localised on nodes of parylene-C could be stimulated with UV laser nanosecond stimulation without extracellular Ca^2+^ and *hypothesised* that localised stimulation of the ER could be a primary factor in producing a Ca^2+^ response. Therefore, in this study, we provided further evidence to support the *hypothesis* of Raos *et al*. [25] that the Ca^2+^ increases stimulated by nanosecond UV laser, resulted in the mobilisation of Ca^2+^ from the intracellular Ca^2+^ stores (ER) causing in a localised increase in Ca^2+^ in the cytoplasm.

### Calcium propagation

For many years, the most intensively investigated mechanism for intercellular Ca^2+^ wave propagation was based on inositol trisphosphate (IP3) diffusion through gap junction pores, which triggers Ca^2+^ release from IP3-sensitive Ca^2+^ stores in neighbouring cells. However, recent studies have demonstrated that ATP was an important extracellular messenger in propagating Ca^2+^ waves in many cell types. Previous researchers have investigated the role of ATP in astrocyte Ca^2+^ signalling. Takano *et al*. and Lee *et al*. [37, 39] observed that mechanically stimulated Ca^2+^ waves in purified cortical primary astrocytes from mice could propagate to neighbouring unconnected astrocytes, which suggested that the signalling pathway was extracellular diffusion of the signalling molecule. After adding phosphate−6-azophenyl-2‘,4‘-disulfonate (PPADS), a purinergic receptor antagonist, Takano *et al*. [39] observed that Ca^2+^ wave propagation did not propagate to neighbouring non-connected astrocyte areas, which suggested that intercellular Ca^2+^ wave propagation was mediated by ATP. Zhao *et al*. [27] have also observed that although IR femtosecond laser-induced Ca^2+^ waves of primary rat astrocytes were significantly blocked by ATP receptor antagonists, but were not blocked by gap junction blockers. In this work, we demonstrated that the propagation of Ca^2+^ wave was significantly impeded by the ATP receptor antagonist, suramin. It was found that chemical blocking of gap junctions did not significantly affect the Ca^2+^ wave propagations. These results indicated that ATP, rather than gap junction channels, were responsible for mediating the transmission of laser-induced intracellular Ca^2+^ signalling in hNT astrocytes which occurred at the neighbours and next nearest neighbour regions in organised networks.

However, various studies have reported the contribution of gap junction to astrocytic Ca^2+^ propagation. Hill *et al*. [30] observed that Ca^2+^ propagation region of mechanically stimulated NT2 astrocytes in non-patterned well-plates was reduced both by a gap junction blocker, CBX, and a purinergic receptor antagonist, PPADS. Thus, Hill *et al*. [30] demonstrated the Ca^2+^ propagation was at least partly dependent on gap junction communication and mediated by the ATP. Fujii *et al*. [31] also demonstrated that Ca^2+^ waves of mechanically stimulated rat primary astrocytes on glass coverslips propagated proximally via gap junctions and distally via extracellular diffusion of ATP. Although the Ca^2+^ propagation inhibited by gap junction blockers was not measured to be highly significant, several studies have demonstrated that blocking gap junction might slightly inhibit the ATP release. For example, Cotrina *et al*. [46] demonstrated that, in rat primary astrocytes, connexin expression enhanced the release of ATP. Stout *et al*. [47] reported that ATP could also be released through connexin hemichannels in rat primary astrocytes, which subsequently participate in intercellular Ca^2+^ communication. Thus, we suggested that in hNT astrocytes, the Ca^2+^ wave is mainly mediated by the extracellular ATP and partially collaborated with gap junctions.

We observed that the magnitudes of the Ca^2+^ responses of neighbouring cells were significantly lower than that of the stimulated cells. This could be attributed to different mechanisms of Ca^2+^ production in the stimulated astrocytes and neighbouring astrocytes. In this work, we demonstrated that Ca^2+^ increases in laser-stimulated cells were dependent on intracellular Ca^2+^ storage, which can then be subsequently amplified by the CICR. The Ca^2+^ responses of neighbouring cells are mainly dependent on ATP binding to the membrane-bound receptors, resulting in the generation of IP3 which triggers the release of intracellular Ca^2+^ from Ca^2+^ stores. In addition, there can be ATP drift due to the fluid dynamics of the media, which affected the ATP diffusion and how much ATP is available for uptake.

We suggest that the organised chip model approach is a more representative model of astrocytes in the brain albeit in 2D and regular, over the conventional petri dishes culture models where unpatterned hNT astrocytes are typically observed to be a ‘fried egg’ shape and touch all of the adjacent astrocytes at their circumference as shown in Fig 2.

## Conclusion

In this article, we carried out an investigation to identify the underlying initiation and propagation mechanisms of intercellular Ca^2+^ waves in organised 5×5 hNT astrocyte stellate-like networks under UV nanosecond laser stimulation, at the single-cell level, for the first time. First, we demonstrated that Ca^2+^ increases in organised laser stimulated cells were mainly dependent on Ca^2+^ released in Ca^2+^ stores (ER). Furthermore, we demonstrated that the Ca^2+^ propagation from the stimulated cell to neighbouring cells is primarily mediated by extracellular ATP rather than the contributions of gap junctions. This work provides valuable insight, for the first time, on the mechanisms of Ca^2+^ wave initiation and propagation in organised adult human stellate-like astrocytic networks following precision activation, using UV nanosecond laser stimulation, demonstrating that extracellular ATP transmission is responsible for Ca^2+^ release at the local network scale rather than gap junctions; being observed even down to the nearest neighbour and next nearest neighbouring cells—contrary to the reported gap junction.

This discovery has significant ramifications to many neurological conditions such as epilepsy, stroke and aggressive astrocytomas where gap junctions can be targeted. In cases where such gap junction targeting has failed, this new finding suggests that these conditions should be re-visited and the ATP transmission pathway targeted instead.
